# Recent advances in PET-MRI for cardiac sarcoidosis

**DOI:** 10.3389/fnume.2022.1032444

**Published:** 2022-12-19

**Authors:** Camila Munoz, Alina Schneider, René M. Botnar, Claudia Prieto

**Affiliations:** ^1^ School of Biomedical Engineering and Imaging Sciences, King’s College London, London, United Kingdom; ^2^ Escuela de Ingeniería, Pontificia Universidad Católica de Chile, Santiago, Chile; ^3^ Millenium Institute for Intelligent Healthcare Engineering iHEALTH, Santiago, Chile; ^4^ Instituto de Ingeniería Biológica y Médica, Pontificia Universidad Católica de Chile, Santiago, Chile

**Keywords:** cardiac sarcoidosis, hybrid PET-MR, whole-heart imaging, quantitative MRI, T1/T2 mapping

## Abstract

The diagnosis of cardiac sarcoidosis (CS) remains challenging. While only a small fraction of patients with systemic sarcoidosis present with clinically symptomatic CS, cardiac involvement has been associated with adverse outcomes, such as ventricular arrhythmia, heart block, heart failure and sudden cardiac death. Despite the clinical relevance of having an early and accurate diagnosis of CS, there is no gold-standard technique available for the assessment of CS. Non-invasive PET and MR imaging have shown promise in the detection of different histopathological features of CS. More recently, the introduction of hybrid PET-MR scanners has enabled the acquisition of these hallmarks in a single scan, demonstrating higher sensitivity and specificity for CS detection and risk stratification than with either imaging modality alone. This article describes recent developments in hybrid PET-MR imaging for improving the diagnosis of CS and discusses areas of future development that could make cardiac PET-MRI the preferred diagnostic tool for the comprehensive assessment of CS.

## Introduction

Sarcoidosis is a multisystemic disorder characterized by the formation of compact non-necrotizing granuloma, mainly affecting the lungs, but that can develop in the skin, eyes, liver, lymph nodes, heart, spleen, and any other organ (1). Sarcoidosis resolves spontaneously in about 50% of patients within two years, but it might become chronic in some patients and develop into chronic inflammation and fibrosis in the affected organs (2). Cardiac involvement is clinically manifested in about 5% of patients with systemic sarcoidosis, however, autopsy and non-invasive imaging studies suggest that the true prevalence of cardiac sarcoidosis (CS) is at least 25%, with the heart being the only organ affected by the disease in up to one-third of cases (3). Patients with CS have poorer outcomes compared to patients with sarcoidosis without cardiac involvement, with higher rates of ventricular arrhythmias, high-degree heart block, heart failure and sudden cardiac death. Sudden cardiac death is in fact the leading cause of death among patients with CS and is often the first manifestation of the disease (4).

Despite its clinical relevance, the diagnosis of CS remains a challenge. Positive endomyocardial biopsies can provide a definite diagnosis, but the sensitivity of this technique is low (<25%) due to the patchy nature of the disease (3). Therefore, diagnosis usually relies on the patient's clinical history together with advanced non-invasive cardiac imaging techniques, including magnetic resonance imaging (MRI) and positron emission tomography (PET). Indeed, while still requiring evidence of biopsy-proven extracardiac sarcoidosis, international guidelines for the diagnosis of CS have recently incorporated cardiac MRI or PET abnormalities as major diagnostic criteria (5, 6). Abnormal findings in MRI and PET reflect different histopathological features of CS. In cardiac MRI, for instance, late gadolinium enhancement (LGE) imaging can be used to assess the presence of fibrotic areas in the myocardium, while ^18^F-fluorodeoxyglucose (^18^F-FDG) PET can be used for detecting areas of active inflammation.

The recent introduction of integrated PET-MRI scanners has shown promise for the comprehensive assessment of CS from a single examination (7, 8). This mini-review focuses on the advantages of such an approach considering recent technical developments and discusses the potential role of simultaneous PET-MRI for improving the diagnosis of CS.

## Diagnosis of CS: role of PET and MR imaging

The histopathological changes throughout the development of CS relate to the findings that are usually observed with each imaging modality. This section briefly summarizes the main histopathological features of each stage of CS, focusing on the complementary roles of PET and MRI in the detection and characterization of the disease (Table 1).

**Table 1:** Main histopathology features in the different patterns of cardiac sarcoidosis and corresponding potential PET and MR imaging findings.

Histopathological features	^18^F-FDG PET	MRI T2w imaging or T2-mapping	MRI LGE or T1-mapping
Lymphocytic infiltration, tissue edema, scattered granuloma	Abnormal	Possibly abnormal	Normal
Well-formed granulomas, varying degrees of fibrosis	Abnormal	Possibly abnormal	Possibly abnormal
Myocardial scarring, fibrosis	Mostly normal	Normal	Abnormal

CS can affect any structure in the heart, including the coronary arteries, pericardium, and valves. However, the myocardium is the structure most frequently affected, with granulomas most often found in the basal segments of the interventricular septum, and the inferior wall of the left ventricle (9). In the early stages of the disease, the main histological features are lymphocytic infiltration, with some tissue edema and scattered granuloma formation. While this stage is usually clinically silent, tissue abnormalities might be detected by using ^18^F-FDG PET, to assess myocardial inflammation, or T2-weighted [and more recently, T2-mapping (10)] MR, to detect myocardial edema. CS may then progress to the accumulation of granulomas in larger areas of the myocardium, with varying degrees of fibrosis. Inflammation is still present at this stage, and therefore abnormalities in ^18^F-FDG PET may still be found, while patchy fibrosis can result in abnormal LGE and T1-mapping findings in cardiac MRI. These stages are sometimes referred to as “active” CS. Finally, in more advanced stages, the disease may progress to tissue scarring and extended areas of fibrosis in the left ventricle myocardium, with thinning of the basal septum. Most inflammation has subsided at this point and therefore, ^18^F-FDG PET images are mostly normal, while positive findings can be found by LGE and T1-mapping cardiac MRI. This stage is usually referred to as “chronic” or “burned out” CS (9).

A recent meta-analysis review compared the accuracy of cardiac MRI and ^18^F-FDG PET for the diagnosis of CS, including nearly 2,000 patients from 33 studies (11). Using as a reference criterion either the Japan Ministry of Health and Welfare (JMHW) criteria (12), the Heart Rhythm Society criteria (5), the Japanese Circulation Society criteria (6), or histological confirmation of the disease when available, a total of ∼690 patients presented with CS. Results showed that cardiac MR had a significantly higher sensitivity than ^18^F-FDG PET (95% vs. 84%), while there were no statistically significant differences in specificity (85% vs. 82%). Furthermore, a more detailed analysis of the studies using PET imaging showed that quantitative evaluation of ^18^F-FDG, i.e., using standardized uptake value (SUV) measurements as criteria for diagnosis of disease, resulted in significantly higher sensitivity than qualitative assessment of the images (i.e., visual detection focal or focal-on diffuse uptake) (93% vs. 76%), without affecting specificity. On the other hand, in cardiac MR, sensitivity was significantly higher in studies that included both LGE and T2-weighted imaging, compared to studies where LGE only was used (99% vs. 88%). This study did not distinguish between active and chronic sarcoidosis and included both untreated and treated patients, which might affect the sensitivity of each technique. Nevertheless, it shows the potential of each of these imaging modalities for an accurate diagnosis of CS.

## Hybrid PET-MR imaging in CS

The ability of PET and MRI to provide complementary information about CS means that, in practice, patients might undergo both examinations to fully characterize disease activity, inform decisions in patient management, and monitor therapy response (13, 14). Hybrid PET-MR imaging offers an efficient way of acquiring this information from a single scan, with the additional advantage of enabling the direct fusion of the images obtained by the two techniques to improve clinical interpretation (15). Furthermore, while guidelines for standardized patient preparation have recently been introduced to minimize potential false-positive ^18^F-FDG PET uptake (16), unsuccessful patient preparation can affect up to 25% of patients (17), and in such cases, hybrid PET-MRI might improve confidence in the diagnosis by providing additional information.

Most studies in hybrid PET-MR imaging for the assessment of CS, including detection and characterization of the disease and prediction of adverse events, have focused on combining ^18^F-FDG PET and cardiac LGE MRI alone (18–20). Based on this approach, four distinct patterns have been observed (21): (1) MRI-positive/PET-positive, where a non-ischemic LGE pattern is aligned with focal or focal-on-diffuse ^18^F-FDG uptake, likely representing active disease; (2) MRI-positive/PET-negative, characterized by the presence of LGE but with no increases in ^18^F-FDG uptake, probably representing chronic disease with myocardial scarring; (3) MRI-negative/PET-negative, with no presence of LGE nor increase of ^18^F-FDG uptake in the myocardium, likely reflecting absence of CS; and (4) MRI-negative/PET-positive, where either focal, focal-on-diffuse or diffuse patterns of increased ^18^F-FDG uptake are observed in absence of corresponding LGE findings. The latter pattern has been associated with incomplete physiological suppression of ^18^F-FDG uptake, but it might also represent true myocardial inflammation in the early stages of the disease where tissue fibrosis is still not present.

Wisenberg et al. (20) compared hybrid PET-MR and PET-CT images acquired on the same day, including 10 patients with known or suspected CS, and found similar patterns of ^18^F-FDG uptake for both scans. A diversity of patterns of disease was observed in this cohort, with most patients (40%) presenting a chronic sarcoidosis pattern of negative ^18^F-FDG findings in presence of scarring detected by LGE MRI, indicating the added value of the hybrid PET-MR examination. Interestingly, one of the patients presented a pattern of MRI-positive/PET-positive in unmatched anatomical locations, but no potential explanation was provided by the authors in terms of possible failed myocardial suppression in the PET images.

A larger cohort of 51 patients was studied by Wicks et al. (19), 33 of which had CS according to the JMHW criteria, demonstrating that hybrid PET-MRI provides an increased sensitivity (94%) compared to either PET or MR alone (85% and 82%, respectively). Patients were followed up for a median of 2.2 years, and authors found that the presence of abnormalities on both PET and MRI was the strongest predictor of major adverse cardiac events (MACE) when compared to abnormalities only in PET or MR, suggesting that hybrid PET-MRI also offers prognostic value. Finally, there was poor agreement between imaging modalities for the regional distribution of ^18^F-FDG and LGE, potentially reflecting the heterogeneity in disease development within the myocardium at the time of imaging.

A study focusing on the ability of hybrid PET-MRI for differentiating between active and chronic CS in 25 patients is presented by Dweck et al. (18). Using the patterns of disease described above to classify patients into active CS (LGE and ^18^F-FDG positive), chronic CS (LGE-positive/^18^F-FDG negative), no CS (LGE and ^18^F-FDG negative) and inconclusive results (LGE-negative/^18^F-FDG-positive). Authors found 8 patients with active disease, one patient with chronic disease, and 8 normal studies. When further analyzing the inconclusive cases, 6 patients (24%) exhibited diffuse ^18^F-FDG uptake, likely indicating a false positive result due to incomplete myocardial suppression. Further two cases showed focal or focal-on-diffuse uptake, which might reflect the detection of an earlier stage of the disease.

One of the limitations of the above studies is the lack of T2-weighted MRI in the analysis of hybrid PET/MR data. Focal hyperintensity in T2-weighted images is associated with tissue edema and might improve confidence in discriminating true myocardial inflammation from false positive results in LGE-negative/^18^F-FDG-positive cases. An early study by Hanneman et al. (22) in 10 patients defined MRI-positive as either the presence of LGE or focal increases in signal in T2-weighted images. Authors found that LGE was present in 66.7% of patients, while T2-weighted hyperintensity was present in 50% of patients. However, when analyzing results at a myocardial segment level, hyperintensity in T2- weighted images and presence of LGE matched only in two myocardial segments from a single patient. Furthermore, they found that by combining ^18^F-FDG PET and MR findings, a sensitivity of 100% could be achieved in this small cohort of patients, which was higher than MR (80%) or PET (90%) alone.

T2-weighted imaging has shown promise for improving the sensitivity of MR to the early stages of CS; however, images are susceptible to motion and artefacts, and therefore, are not always included in routine clinical examinations. More recently, quantitative T2-mapping techniques have been shown to provide an objective assessment of myocardial inflammation (10, 23, 24) with increased robustness and reproducibility. In addition, quantitative T1-mapping techniques have been progressively adopted by the cardiac MR community for the assessment of diffuse fibrosis (25–27). These techniques have been recently demonstrated in the context of hybrid PET-MR imaging for CS (28, 29), where authors propose a multi-parametric approach including T1-mapping, T2-mapping, and LGE cardiac MR plus ^18^F-FDG PET for a comprehensive diagnosis and prognostication of the disease.

The study by Greulich et al. (28) included 43 patients with biopsy-proven extracardiac sarcoidosis and focused on the differentiation between active and chronic CS. In this study, cardiac MR findings (i.e., presence of LGE and/or elevated T1 and T2 values in the myocardium) were used to diagnose CS, while PET findings were used to classify patients into active disease, if there was focal or focal-on-diffuse increased myocardial ^18^F-FDG uptake, or chronic disease in absence of ^18^F-FDG abnormalities. In contrast to previous studies, no CS was defined as normal cardiac MR regardless of PET findings. Seven patients were excluded due to unsuccessful suppression of myocardial ^18^F-FDG uptake, while 13 patients presented with active sarcoidosis, 5 with chronic disease and 18 patients were considered to not have CS. Interestingly, CS was diagnosed based on elevated T1 values in four patients who had negative LGE findings, highlighting the added value of including quantitative MR mapping techniques in the assessment of CS.

**Figure 1:**
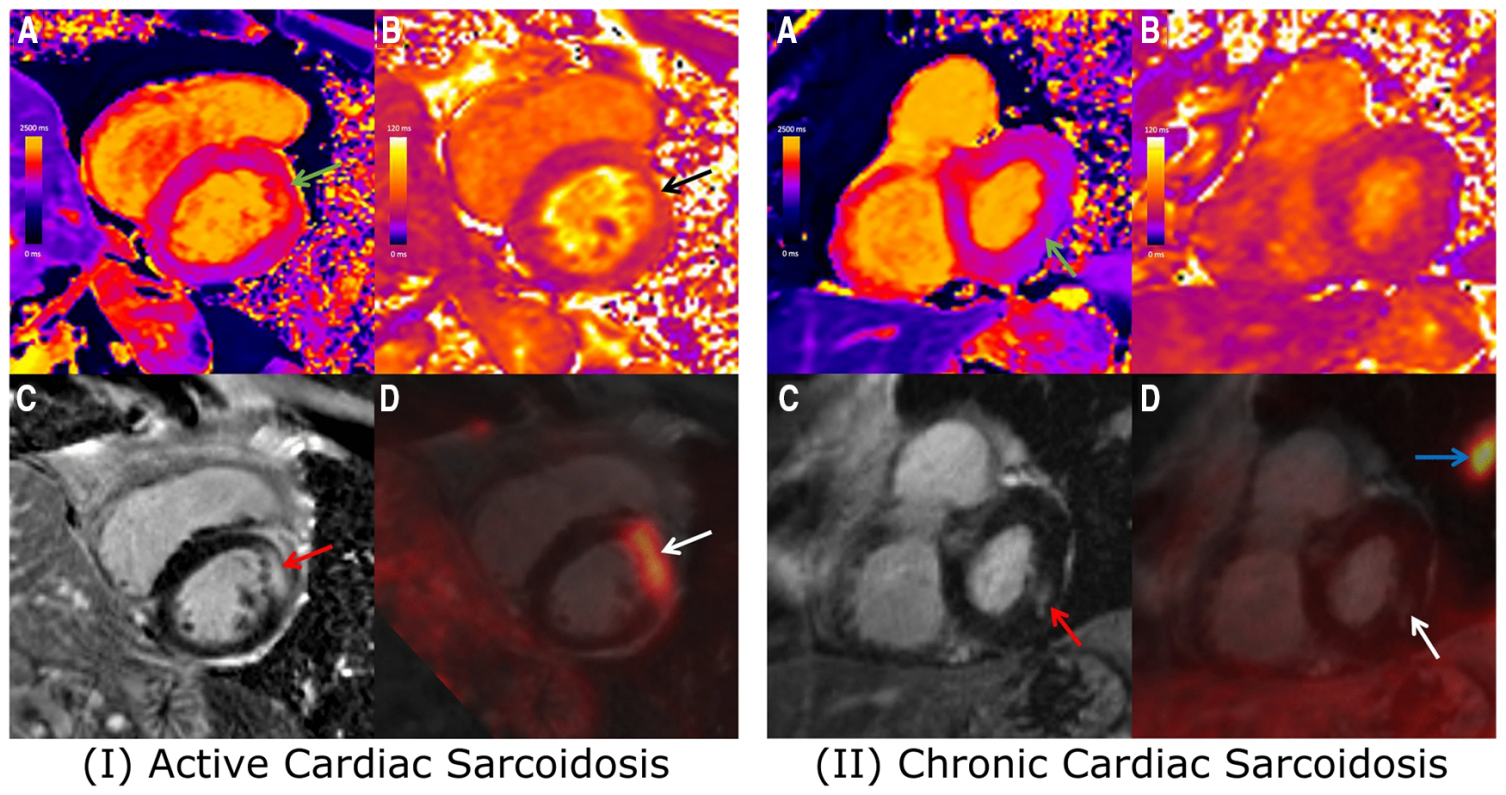
Combined ^18^F-FDG PET/MRI images showing short-axis slices including (**A**) native T1 map, (**B**) native T2 map, (**C**) LGE image, and (**D**) fused ^18^F-FDG PET and LGE image. (**I**) 67-year-old Male with cardiac and extra-cardiac sarcoidosis, with co-localized elevated T1 (green arrow), elevated T2 (black arrow), mid-wall LGE (red arrow) and focal FDG uptake (white arrow). With positive findings on both PET and MRI, the patient likely presents with active cardiac sarcoidosis. (II) 72-year-old Male with cardiac and extra-cardiac sarcoidosis, demonstrating slightly elevated T1 (green arrow) and corresponding mid-wall LGE (red arrow) at the inferolateral wall. No corresponding elevation of T2 or focal FDG uptake (white arrow) was observed, likely reflecting chronic, burnt-out cardiac sarcoidosis. *Adapted from Cheung, E., et al. (2021). Combined simultaneous FDG-PET/MRI with T1 and T2 mapping as an imaging biomarker for the diagnosis and prognosis of suspected cardiac sarcoidosis. European Journal of Hybrid Imaging*, *5*(1) and the original ahs a CC BY 4.0 license, available here: https://ejhi.springeropen.com/articles/10.1186/s41824-021-00119-w#rightslink.

Cheung et al. (29) used a similar hybrid ^18^F-FDG PET with LGE, T1 mapping and T2 mapping MRI approach for the diagnosis and prognosis of CS in 42 patients who were followed up for a median of 1.7 years (Figure 1). Findings showed that presence of LGE and elevated T1 values were the criteria with the highest sensitivity for diagnosing CS (100% in each case), while the highest specificity was provided by focal ^18^F-FDG uptake (69%) and elevated T2 values (79%). Overall, the presence of focal ^18^F-FDG uptake colocalized with LGE or elevated T1 provided the highest diagnostic performance (73%). Results also confirmed the findings of Wicks et al. (19) in terms of the prognostic value of hybrid PET-MR, with patients exhibiting a co-localized pattern of ^18^F-FDG uptake and LGE or elevated T1 having a 12-fold increase in risk of MACE compared to patients without such findings.

## Opportunities and challenges

As discussed above, hybrid PET-MRI has shown promising results for the assessment of CS. However, challenges remain for the widespread adoption of this technique. Most clinical cardiac MRI protocols rely on repeated breath-holding and electrocardiogram triggering to produce images free of respiratory- and cardiac-induced motion artefacts, limiting the available time for data acquisition and resulting in images with limited volumetric coverage and spatial resolution. In practice, this means that cardiac MR images are usually acquired as a series of 2D images with different geometries and orientations, i.e., 2-chamber, 3-chamber, 4-chamber, and stacks of short-axis views, with a large slice thickness (8 to 10 mm), which may hinder the depiction of small patchy lesions. On the other hand, cardiac PET imaging is intrinsically a 3D technique, and clinical protocols rarely incorporate motion compensation techniques, resulting in motion-blurred images and leading to misaligned cardiac PET and MR images that can be difficult to interpret together. Two major technical innovations in cardiac MR and PET-MR imaging that may alleviate these issues are whole-heart 3D MR and motion-corrected PET-MR imaging techniques.

3D whole-heart MR can provide higher spatial resolution and increased volumetric coverage compared to multi-slice multi-breath hold 2D imaging. However, such an approach requires longer scan times and therefore motion compensation and/or accelerated data acquisition techniques are fundamental to making 3D cardiac MRI clinically feasible. A great variety of techniques have been introduced in cardiac MR to enable whole-heart imaging for different cardiac applications, and a comprehensive review is out of the scope of this article. Instead, we will briefly discuss some of the techniques that have been developed for 3D LGE, 3D T1-mapping and 3D T2-mapping, given their relevance to the diagnosis of CS.

Several 3D LGE imaging techniques have been proposed in the literature that rely on respiratory motion-compensation mechanisms to enable free-breathing acquisitions with isotropic or near-isotropic spatial resolution (30–33). In combination with parallel imaging, these approaches have been shown to achieve voxel sizes of about 2 mm isotropic, from scans ranging from 4 to 15 min. Alternatively, some approaches have used accelerated data acquisition to achieve single breath-hold imaging (34–37). While enabling significantly shorter scans, these techniques require breath-holds of ∼20s that might be difficult to achieve for patients. Furthermore, due to limitations in the available scan time, the single breath-hold approach can only achieve a limited spatial resolution, with a typical slice thickness of 5 to 10 mm.

Similar efforts have been made to move toward 3D T1- and T2-mapping. Most whole-heart T1-mapping approaches have used respiratory triggering techniques, whereby monitoring respiratory motion and only accepting data acquired during end-expiration, respiratory-induced motion artefacts can be significantly reduced. One drawback of this approach is that as the remainder of the respiratory cycle is excluded from data acquisition, the scan time significantly lengthens. To achieve a clinically feasible scan time of 6–10 min, most 3D T1 mapping approaches have used non-isotropic spatial resolution, with 1.2–1.7 mm in-plane resolution but 4 to 16 mm slice thickness (38–41). Similarly, for 3D T2-mapping, most approaches have relied on thick slices (5–6 mm) to produce whole-heart with around 10 min scan time (42, 43). 3D T2-mapping with a high isotropic resolution of 1.7 mm was introduced by Van Heeswijk et al. (44), however, long scans of ∼18 min prevented its adoption in clinical practice.

More recently, higher data acceleration in combination with more advanced motion compensation techniques has enabled the acquisition of whole-heart datasets with high isotropic spatial resolution from clinically feasible scan times. 3D LGE can now achieve isotropic resolution of 1.3–1.4 mm from ∼7 min scans (45–47), while 3D T1- and T2-mapping techniques with 1.5–1.6 mm isotropic resolution from ∼10 min scans have been recently demonstrated (48–50). These approaches are promising for the detection of smaller patchy lesions which might be present in CS.

Another area where hybrid PET-MR scanning has the potential of improving the diagnosis of CS is MR-based motion correction. By acquiring MR images that can provide respiratory and cardiac motion information, the simultaneously acquired PET data can be corrected for motion, resulting in improved delineation and quantification for cardiac PET images. A review of these techniques can be found in (51). While most of these techniques have focused on myocardial viability imaging with ^18^F-FDG PET, the study by Robson et al. (52) studied the potential of MR-based cardiac and respiratory motion correction in patients with CS, demonstrating that this approach resulted in improved visual appearance of the ^18^F-FDG uptake pattern in areas of the heart most affected by motion, and in increased contrast of the detected lesions. More recently, Schneider et al. (53) has demonstrated the technical feasibility of a 3D T2-mapping technique that provides respiratory motion information, enabling simultaneous motion-corrected ^18^F-FDG PET and whole-heart T2-mapping. The clinical impact of such technique remains to be studied.

Finally, while most studies using PET-MR for the diagnosis of CS have used ^18^F-FDG to assess myocardial inflammation, alternative radiotracers that target somatostatin receptors in sarcoid granulomas may provide higher specificity for the detection of inflammatory or proliferating cells, including gallium-68 (Ga-68) DOTATOC, Ga-68 DOTATATE, and Ga-68 DOTANOC (9, 21). Because of their specificity, these tracers do not require specific patient dietary or fasting preparation, showing promise for simplified cardiac PET-MR examinations.

## Outlook

Hybrid PET-MR has shown potential for improved diagnosis, assessment of disease activity, therapy response monitoring and prognostication in cardiac sarcoidosis from a single comprehensive scan. These two imaging modalities provide complementary information that can improve diagnosis sensitivity in early sub-clinical stages of the disease and can guide patient management decisions.

Recent technical innovations in cardiac MR and PET-MR have demonstrated a variety of solutions for cardiac MR to achieve high isotropic spatial resolution with whole-heart coverage, and mechanisms to prevent image degradation in PET images due to physiological motion, resulting in improved image quality and quantification, and potentially increasing diagnostic confidence and accuracy. Further studies about the clinical impact of such improvements are now needed to evaluate the added value of hybrid PET-MR for the comprehensive evaluation of cardiac sarcoidosis.
